# Comparing Smartphone Virtual Reality Exposure Preparation to Care as Usual in Children Aged 6 to 14 Years Undergoing Magnetic Resonance Imaging: Protocol for a Multicenter, Observer-Blinded, Randomized Controlled Trial

**DOI:** 10.2196/41080

**Published:** 2023-01-24

**Authors:** Zita van Spaendonck, Koen Pieter Leeuwenburgh, Marjolein Dremmen, Joost van Schuppen, Daniëlle Starreveld, Bram Dierckx, Jeroen S Legerstee

**Affiliations:** 1 Department of Child and Adolescent Psychiatry/Psychology Erasmus University Medical Center Sophia Children’s Hospital Rotterdam Netherlands; 2 Department of Radiology Erasmus University Medical Center Sophia Children’s Hospital Rotterdam Netherlands; 3 Department of Radiology and Nuclear Medicine Amsterdam University Medical Centers Emma Children’s Hospital Amsterdam Netherlands

**Keywords:** virtual reality, VR, children, anxiety, magnetic resonance imaging, MRI, MRI scans, imaging, randomized controlled trial, MRI preparation, smartphone virtual reality, smartphone intervention, procedural anxiety, psychosocial intervention

## Abstract

**Background:**

A magnetic resonance imaging (MRI) procedure can cause preprocedural and periprocedural anxiety in children. Psychosocial interventions are used to prepare children for the procedure to alleviate anxiety, but these interventions are time-consuming and costly, limiting their clinical use. Virtual reality (VR) is a promising way to overcome these limitations in the preparation of children before an MRI scan.

**Objective:**

The objective of this study is (1) to develop a VR smartphone intervention to prepare children at home for an MRI procedure; and (2) to examine the effect of the VR intervention in a randomized controlled trial, in which the VR intervention will be compared to care as usual (CAU). CAU involves an information letter about an MRI examination. The primary outcome is the child’s procedural anxiety during the MRI procedure. Secondary outcomes include preprocedural anxiety and parental anxiety. We hypothesize that the VR preparation will result in a higher reduction of the periprocedural anxiety of both parents and children as compared to CAU.

**Methods:**

The VR intervention provides a highly realistic and child-friendly representation of an MRI environment. In this randomized controlled trial, 128 children (aged 6 to 14 years) undergoing an MRI scan will be randomly allocated to the VR intervention or CAU. Children in the VR intervention will receive a log-in code for the VR app and are sent cardboard VR glasses.

**Results:**

The VR smartphone preparation app was developed in 2020. The recruitment of participants is expected to be completed in December 2022. Data will be analyzed, and scientific papers will be submitted for publication in 2023.

**Conclusions:**

The VR smartphone app is expected to significantly reduce pre- and periprocedural anxiety in pediatric patients undergoing an MRI scan. The VR app offers a realistic and child-friendly experience that can contribute to modern care. A smartphone version of the VR app has the advantage that children, and potentially their parents, can get habituated to the VR environment and noises in their own home environment and can do this VR MRI preparation as often and as long as needed.

**Trial Registration:**

ISRCTN Registry ISRCTN20976625; https://www.isrctn.com/ISRCTN20976625

**International Registered Report Identifier (IRRID):**

DERR1-10.2196/41080

## Introduction

Magnetic Resonance Imaging (MRI) is a commonly used imaging technique that provides detailed images without the use of ionizing radiation. Research has shown that pediatric patients can experience a range of negative emotions during this procedure [1]. There are several components within an MRI procedure that can induce anxiety such as the loud noises generated during an MRI scan and the prolonged time spent in a confined space [2]. Although there is little research on how children experience an MRI scan, one study found that about 50% of children between aged 5 to 12 years with nonacute medical conditions experienced anxiety and discomfort during an MRI scan [3]. Younger children can be overwhelmed by the MRI environment with noises they have never heard before and by the MRI machine, whereas older children can experience claustrophobic feelings [4]. This can result in poor cooperation, eg, following breathing instructions or excessive movements, which has a negative effect on the scan quality since up to 90% of the MRI artifacts can be attributed to movement [5]. To combat MRI-related anxiety and discomfort, children are often sedated or completely anesthetized up to the age of 6 years [6,7]. The use of general anesthesia is not without risks. Pediatric patients can experience side effects such as emerging delirium, respiratory depression, nausea, vomiting, agitation, and cardiovascular bradycardia [8-11]. Different methods have been implemented to reduce the need for anesthesia and pre- and periprocedural anxiety, such as behavioral rehearsal using mock MRI scans [12], play therapy [13], and distraction during the procedure using MRI-compatible audiovisual tools [14]. The use of these interventions has been found to be effective but are time-consuming and often rely on the guidance by a professional [2].

To alleviate MRI-related anxiety, virtual reality (VR) offers a promising alternative to prepare children for the procedure. VR is a way to experience computer-generated scenarios that can be used to create experiences similar to or completely different from reality. With audiovisual stimulation, the user can interact with the virtual environment [15]. Research has shown that VR can positively affect self-reported pain and anxiety during several medical procedures [16]. Most VR studies focused on distracting patients during a medical procedure. Exposure, however, is one of the most effective interventions to reduce anxiety, and only a few studies investigated the impact of VR exposure before a medical procedure [17-20].

To our knowledge, there has been little research into the effectiveness of VR as preparation for an MRI in a clinical setting. In a cross-sectional study of 23 pediatric patients by Ashmore and colleagues [21], the VR preparation was highly rated on enjoyment, helpfulness, and ease of use. They also showed that for some pediatric patients, VR preparation helped them to undergo an MRI scan without general sedation. In an exploratory study, Nakarada-Kordic and colleagues [22] compared the feeling of being inside an MRI scanner in VR versus being in a mock MRI scanner in a sample of 20 adult participants. The authors did not find a significant difference between the experience of VR MRI and mock MRI scans, and 86% of the participants reported that the VR simulation could be a helpful way to prepare for a real MRI exam. Stunden and colleagues [19] studied VR exposure based on a 360-degree movie versus a standard preparatory manual and a hospital-based Child Life Program on anxiety in a simulated MRI scan in 92 children aged 4-13 years but could not demonstrate a difference between the 3 groups. While this study failed to find a significant effect of VR exposure on procedural anxiety, it did have several drawbacks. They used a simulated MRI scan instead of an actual clinical MRI scan to test intervention effectivity. This may have limited the emotional response generated. Finally, the use of 360-degree recorded images instead of rendered 3D graphics may have limited immersion, which could be an important mediator of intervention effectivity as shown in VR-based pain distraction [23].

VR preparation of pediatric patients for medical procedures is often done with a head-mounted displays tethered to a powerful computer within the hospital setting. A disadvantage of this approach is that it requires a trained professional to do the VR preparation and that the time a child can be prepared is often limited. An alternative is to deploy the VR preparation to a smartphone, head-mounted by means of a Google cardboard. With a smartphone VR app, children can get prepared in their own home environment under parental supervision at their own pace and as often and as long as they need. Another advantage of a smartphone VR app is that it can be employed on a large scale and requires limited involvement of health professionals, ultimately saving costs associated with the traditional VR goggles tethered to a powerful computer. Moreover, costs associated with a Google cardboard are negligible compared with computer-tethered or dedicated head-mounted VR displays.

The objective of this study is (1) to develop a realistic VR smartphone app to prepare children for an MRI scan, using an immersive, interactive VR approach with a fully rendered environment; and (2) to present a methodological approach to conduct a randomized controlled trial (RCT) that can test the effectiveness of the VR intervention in children who need to undergo an MRI scan. The clinical effect of the VR smartphone app will be compared with routine clinical care (care as usual [CAU]). The primary outcome is periprocedural anxiety. Secondary outcomes include preprocedural anxiety, user experience, child behavioral and emotional problems, and parental anxiety. We hypothesize that the VR preparation will result in a larger reduction of the periprocedural anxiety of both parents and children as compared to CAU. We further hypothesize that VR preparation will lead to a larger reduction in parental anxiety and preprocedural child anxiety as compared to CAU.

## Methods

### VR Design

The VR tool encompasses a highly realistic virtual environment that replicates the MRI experience of the Erasmus University Medical Center (MC)–Sophia Children’s Hospital, Rotterdam, the Netherlands. The designed virtual MRI environment does not significantly differ from that of the Amsterdam University Medical Centers (UMC).

A multidisciplinary team, consisting of child-life specialists, child psychologists, a child psychiatrist, anesthesiologists, a 3D-acting director, and a 3D project manager designed the script of the VR experience. Working together with specialized VR developers and animators, multiple 3D characters, asset, and environment artists created the scenery and character modeling. During the design and development phases, team meetings were held to review the process and make any necessary adjustments. Overall, the main goal was to create a dynamic and interactive environment that will prepare children for the MRI procedure in a realistic and child-friendly manner. Once the VR software was created, it was pilot-tested in healthy children (n=10). Based on the observations and responses of the pilot test, final adjustments were made.

### Technical Specifications

#### Software and Hardware Details

The 3D software in the VR smartphone app was developed using the Unity games engine, a cross-platform game engine developed by Unity Technologies. The app is designed to be used in a Google cardboard–like VR goggle, meaning a VR goggle relying on the use of a smartphone for its VR capabilities. The cardboard includes adjustable lenses and head straps—this makes the cardboard suitable for children. The software is usable on iPhone 5s or above or Android smartphones with operating system 4.4 or above. Participants can download the app in the Apple App Store or Google Play Store.

#### VR Storyline

**Figure 1:**
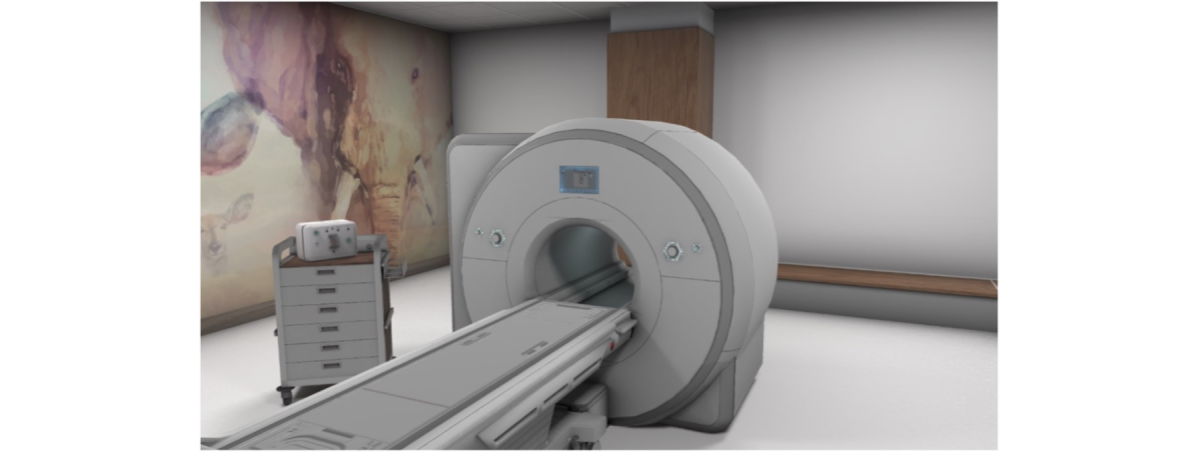
Image of the 3D environment in which the virtual reality experience takes place.

The duration of the VR MRI smartphone intervention is approximately 5 minutes. The VR app takes place in a typical MRI room as seen in Figure 1 (and Multimedia Appendix 1), with an explanation given to a child by MRI technicians depicted on a monitor in the virtual MRI room. This design has been chosen to maximize the ease of program portability between different hospital settings and even between different languages by making it easy to switch out the supporting video with a localized alternative.

Participant can choose between 2 versions: one version for an upper body MRI and another version for a lower body MRI. The MRI experience starts with a technician that is visible on a screen on the wall of the MRI room. The technician introduces herself and explains that the participant is in the MRI room, what an MRI scan is, and that it makes a lot of noise but is not painful. She also explains that no metals are allowed in the MRI environment and that participants are not allowed to wear jewelry during the actual MRI examination. After this initial explanation, the participant is allowed to move around the virtual MRI room. The MRI room and machines are visible in 360 degrees and can be seen if the participant moves with his head. The participant can move around the MRI machine by looking at marked focus points (feet symbol) on the floor of the MRI room. When the participant is located behind the MRI machine, the technician explains that the MRI bore is open from the front to the back. When the participant is back at the starting position in the front of the MRI machine, the technician explains that some children like their parents to be present during the MRI, that they will get earplugs because of the MRI noise, and that it is important that they lie still within the MRI machine. After that, the participant can look at a focus point on the ceiling above the table of the MRI machine and is then transferred to this location. Subsequently, the participant is slowly transferred within the bore of the MRI machine. Depending on the version of the MRI machine, the participant is transferred within the bore of the MRI machine headfirst (upper body MRI) or feetfirst (lower body MRI). A scan with the duration of 1 minute is performed accompanied by varying MRI noises. When the scan is completed, the VR experience is finished, and the app returns to the home screen.

### Study Design

This multicenter, single-blinded, RCT is being performed at the Erasmus MC–Sophia by the Departments of Child and Adolescent Psychiatry/Psychology, Radiology, and Pediatric Anesthesiology and at the Amsterdam UMC–Emma Children’s Hospital by the Department of Radiology. Children (N=128) will be randomly allocated to 2 parallel groups: VR smartphone application (n=64) or CAU (n=64). CAU involves an information letter given to the parents that consists of information about the MRI examination, the screening questionnaire that is used, and how to best explain the MRI exam to their child. Randomization will be performed as block randomization with a 1:1 allocation.

### Ethics Approval

The study was approved by the medical ethical committees of the Erasmus MC (NL68075.078.19) and Amsterdam UMC (MEC-2019-0242).

### Inclusion and Exclusion Criteria

Eligible participants are children aged between 6 and 14 years undergoing an MRI scan at the Erasmus MC–Sophia Children’s Hospital or the Amsterdam UMC–Emma Children’s Hospital (2 specialized children’s university hospitals in the Netherlands) in the period between May 2021 and December 2022. Children that meet the following criteria will be excluded from participating: any intellectual disability, inability of parents to read or write in the Dutch language, epilepsy, visual impairments, and the need of general anesthesia during the scan.

### Sample Size

To conduct analysis of covariance (ANCOVA) on the level of periprocedural anxiety at time point (T) 3 (primary outcome) with a Cohen *d* of 0.5, an α of .05 (2-tailed), and a power of .80, a sample size of 128 patients is needed (64 patients per group). Based on earlier studies, we anticipate finding a medium effect size [16].

### Randomization

Participants will be randomly allocated to the intervention (VR) or control group (CAU) with a 1:1 allocation. A computer-generated randomization schedule will be used, stratified by age group (aged 6-10 vs 10-14 years) and the type of MRI (upper body vs lower body).

### Patient Recruitment and Procedure

For the prospective recruitment of patients, in concert with the radiologist, the planned MRIs will be scanned a month ahead for eligible patients. They will be informed about the study by mail or phone. If needed, they will be reminded by phone in the following weeks. Besides the reminder, this will help to overcome doubts, because the staff can answer questions or motivate the patients to participate.

All participants will receive an invitation, information folders, and an informed consent form. Participation is not obligatory, and all data will be anonymized. Parents of all the patients will be asked to sign informed consent. Children aged 12-14 years will also be asked to provide informed assent themselves. Patients aged younger than 12 years will give their verbal permission.

After receiving signed informed consent, the participants will be stratified by the type of MRI scan (head or body scan). Then, they will be randomly assigned to the VR group (n=64) or the CAU group (n=64).

The children assigned to the VR group will receive Google cardboard–like VR glasses and instructions on how to download the VR app on their phone and use the VR intervention at home. They will receive a log-in code to use the smartphone VR app. Adherence will be promoted by giving the participants detailed information about the purpose of the study and how to use the VR preparation app and explaining how they can get help from the contact support staff if there are difficulties with the app or VR glasses.

The allocation sequence was executed by a researcher who is not involved in the assessment phase. The psychologist who assesses the child’s anxiety state is blinded to the group assignment. This way, blinding for the treatment allocation will be guaranteed. Parents and children in both groups will be asked not to discuss their treatment allocation with the nurse, radiologist, or research psychologist.

Questionnaires will be assessed at the following time points: (1) T1, the weeks before the MRI scan (at home); (2) T2, the day of the MRI scan, before the procedure will start (in the waiting room); and (3) T3, right after the MRI scan (in the waiting room). The T3 questionnaires will retrospectively assess the child’s experience during the MRI scan. In this way, we can measure the anxiety during the MRI scan without the interruption of the procedure.

The children assigned to the CAU group will receive an information letter in which child-friendly information is provided about the VR examination.

### Participant Withdrawal

Participants can withdraw from the study for any reason at any time if they wish to do so, without any consequences. The investigator can decide to withdraw a participant from the study for urgent medical reasons.

### Outcome Measures

**Figure 2:**
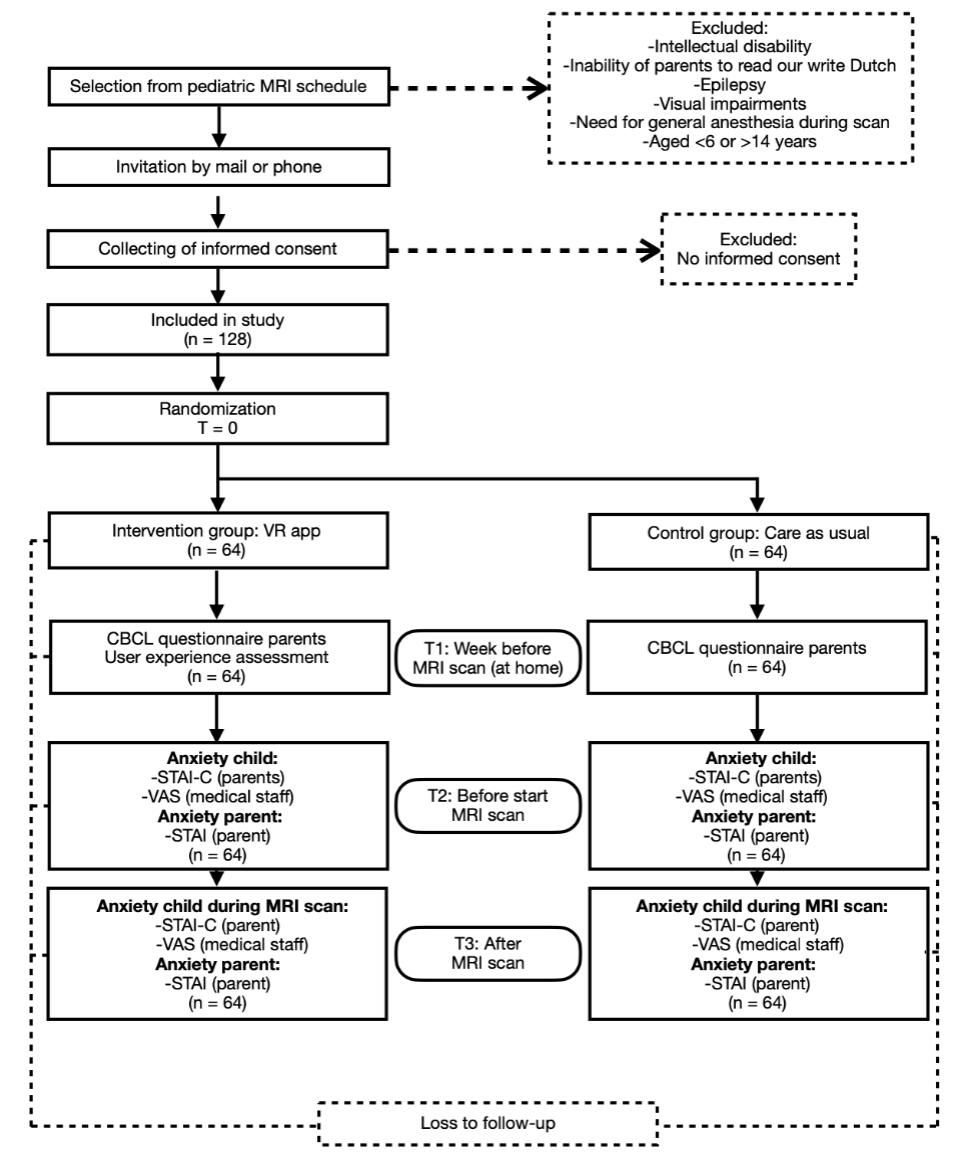
Flow chart of the study design with the instruments at each time point. CBCL: Child Behavioral Checklist; MRI: magnetic resonance imaging; STAI: State-Trait Anxiety Inventory; STAI-C: State-Trait Anxiety Inventory for Children; T: time point; VAS: Visual Analog Scale; VR: virtual reality.

The study design and variables at each time point are presented in Figure 2. The primary outcome is periprocedural anxiety at T3 (State-Trait Anxiety Inventory for Children [STAI-C] and Visual Analog Scale [VAS]). Secondary outcomes include preprocedural anxiety (VAS and STAI-C), user experience, child behavioral and emotional problems (Child Behavioral Checklist [CBCL]), and parental anxiety (State-Trait Anxiety Inventory [STAI]).

#### Background Variables

The following background variables will be collected: age, gender, and socioeconomic status (SES). Age and gender will be collected from the electronic record. SES will be based on a child’s postal code.

#### Child Anxiety

**Figure 3:**
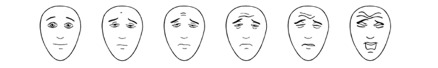
The Visual Analogue Scale (VAS) with faces from not anxious (most left) to extremely anxious (most right).

The anxiety level of participants is measured before and after the MRI scan (T2 and T3) with the STAI-C, a questionnaire where parents and children evaluate statements on a scale from 0 (not at all) to 4 (completely true). The STAI-C is a widely used and validated self-report instrument for measuring state and trait anxiety [24]. The STAI-C has a good-to-excellent interobserver and intraobserver reliability and was validated in Dutch [25]. The VAS will be used to assess the child’s anxiety level by the child himself, the parents, and the medical staff. The VAS shows 6 faces in order from not scared at all (most left face) to extremely scared (most right face; see Figure 3) [26]. The participants select the expression that fits their feeling before and after the MRI scan (T2 and T3). Because of the nonverbal character and the simplicity, this instrument offers a quick and reliable assessment that can be used in different settings [27,28]. The VAS is a widely used and studied instrument, has been shown to have a good reliability and validity, and is validated in Dutch [29]. The medical staff who accompanied the child were instructed to observe the anxiety of the child before the procedure in the MRI room and during the MRI scan (T2 and T3) by using the VAS [30].

#### Child Behavior Problems

In the weeks before the MRI scan (T1), the parents/guardians complete the CBCL to examine behavioral or emotional problems in the last 6 months. The version used for children between the age of 6-18 years consists of 113 items, has been proven valid and reliable, and is validated in Dutch [31]. The respondent evaluates each item as not true (0), a bit/sometimes (1), or obviously/often (2). An internalizing problem and externalizing problem scale can be calculated.

#### Parental Anxiety

Parents/guardians rate their own feelings of anxiety with the STAI at T2 and T3. This self-report instrument contains 2 separate scales for trait and anxiety, combined into 40 items that the respondent rates as not at all (0), somewhat (1), moderately (2), or very much (3). Earlier studies identified the STAI as a validated and reliable instrument to measure the anxiety state of the parents, and it is validated in Dutch [24,32].

#### User Experience

Children will provide subjective assessments of the VR MRI app using a 0-100 graphic rating scale at T1. They are asked (1) to rate the amount of “fun” they had during the VR preparation (no fun to most fun); (2) to what extent they felt nausea as a result of experiencing the virtual world (no nausea to severe nausea/vomiting [VR condition only]); (3) how real the objects in the virtual world seemed (“completely fake” to “indistinguishable from a real object” [VR condition only]); and (4) to what extent they felt like they went into the virtual world while experiencing VR (“I did not feel like I went into the virtual world at all” to “I went completely into the virtual world” [VR condition only]). Adherence will also be addressed in the user experience questionnaire by asking children and parents how often and how long they used the VR preparation app.

### Monitoring and Harms

Monitoring of the study will be done by the Erasmus MC and will take place once a year. During the monitoring, the following will be monitored: (1) study documents and agreements; (2) patient inflow, consent, compliance, and source document verification; (3) patient safety; (4) the investigational intervention (VR); and (5) the study procedures.

The risk associated with the VR intervention is negligible and the burden should be considered minimal.

### Protocol Amendments

Modification to the protocol, which may impact on the conduct of the study and potential benefit of the patient or may affect patient safety, will require a formal amendment to the medical ethical committee of the Erasmus MC.

### Confidentiality

The handling of patient data will comply with the General Data Protection Regulation. All study data will be handled confidentially and coded with a unique study number. Only the research team involved in the study will be able to identify participants and have access to the data.

### Statistical Analysis

#### Overview

The primary analysis will be conducted using an intention-to-treat basis. All patients recruited into the study will be included in this analysis. Patients will be analyzed within the group to which they were randomized, irrespective of what care they received. Regarding missing data, first, it will be analyzed if missing data/loss to follow-up reflects information bias or selection bias. Second, multiple imputation will be used to control for bias due to missing data.

#### Primary Study Parameter(s)

To test the efficacy of the VR intervention, we will compare the VR group to the CAU group with an ANCOVA. The primary outcome variable is the child’s situational anxiety during MRI scan (T3; VAS child, continuous score), corrected for age, sex, baseline anxiety, and CBCL scores as well as type of MRI. In case of nonnormality, we will perform a Mann-Whitney *U* test.

#### Secondary Study Parameter(s)

The main secondary outcomes are child preprocedural anxiety at T2 and parental anxiety both before and during the procedure.

For the child’s preprocedural anxiety, we will employ an identical analysis as for the primary end point. For parental anxiety, a repeated measures ANCOVA will be conducted with parental anxiety at T2 and T3 as within variable and group (VR vs CAU) as between variables. The analysis will be corrected for age, sex, baseline anxiety, and CBCL scores as well as type of MRI.

To identify medical or psychosocial moderators for treatment response to VR, we will perform exploratory linear regression analyses with child anxiety at T2 and T3. Predictor variables that will be examined are SES, age, sex, type of MRI, preprocedural child and preprocedural parental anxiety, and child emotional and behavioral problems. The user experience of children in the VR condition will be examined with descriptive analyses.

## Results

The final version of the VR app has been approved. The recruitment of participants started in May 2021 and is currently ongoing and expected to be completed in December 2022. Data will be analyzed, and scientific papers will be submitted for publication in 2023.

## Discussion

### Expected Findings

Children often experience an MRI scan as an anxiety-provoking situation, and therefore, it is urgently needed that effective psychological preparations that are easy to use and cost-effective are developed and offered. VR has the potential to become a promising tool in the psychological preparation of pediatric patients for MRI. VR exposure has already been shown to be effective as a treatment for specific phobias in children. However, despite the fast-growing field of VR in medical care, there has been little research into the effectiveness of VR as preparation for an MRI scan in a clinical setting. Since VR is a promising tool for improvement in health outcomes, high-quality studies investigating innovative VR interventions are needed. Here, we describe the development of a smartphone-based VR intervention to prepare children for MRI scanning as well as an RCT to test its efficacy.

### Strengths and Limitations

This study design has both strengths and limitations. A strength of this study is the RCT design and the multiple informant assessment of anxiety in children before and during the MRI scan. Most of the current preparation materials for children undergoing an MRI scan involve verbal explanation. The use of VR combines verbal explanation with visual information that can be beneficial for younger children or children who do not speak or understand the language. Additionally, the use of mobile phone–based VR creates the possibility to prepare children in their own time, at their own pace, and in the comfort of their own home. It also alleviates the personnel and space demands imposed by mock-scanner preparation. Due to the use of a smartphone-based VR experience that can be used at home, we can reduce the costs associated with using a tethered VR experience. The app presents a typical MRI room and thus can be used in any hospital without adjustments. Moreover, the app is designed to be easily localized to other languages. Furthermore, due to the use of 3D models instead of 360-degree video recordings, adjustments and adding or subtracting certain elements can be easily and cheaply done.

A limitation is that although we ask participants in the user experience questionnaire how often and for how long they have used the VR preparation app, we do not have more accurate measures of VR use. In addition, as the VR intervention is designed to be used at home in an environment that is comfortable for a child, we do not have information if the children used the VR MRI app with enough attention. The smartphone used by each child varies greatly in screen quality, size, and processing power. These differences might influence the VR experience, its image quality, and ultimately, the immersivity of the VR. Another limitation is that only children who do not need general anesthesia during the MRI scan are included, thereby potentially excluding the most highly anxious children. This may limit the generalizability of this study to children with less severe levels of anxiety. If this study shows that the VR MRI preparation app is efficacious, it would be important to study whether VR preparation can also reduce the need for anesthesia.

### Conclusion

Most children experience anxiety before and during an MRI scan. To reduce this and reduce the need of anesthesia, there is a need to develop a more effective preparation method. VR offers a child-friendly and realistic environment where children can experience the procedure, without the use of real machines or medical staff. To examine the effect of this VR intervention, an RCT will be performed. If VR exposure is proven to be effective for preparation for an MRI scan, this easy-to-use tool can be implemented into standard medical care. We would like to emphasize though that even with the use of modern technology, education provided by health care professionals for both pediatric patients and their parents is still necessary, especially for older children.

## Appendix

Multimedia Appendix 1 Images of the 3D environment in which the VR-experience takes place.

## Abbreviations

ANCOVA: analysis of covariance
Amsterdam UMC: Amsterdam University Medical Centers
CAU: care as usual
CBCL: Child Behavioral Checklist
Erasmus MC: Erasmus University Medical Center
MRI: magnetic resonance imaging
RCT: randomized controlled trial
SES: socioeconomic status
STAI: State-Trait Anxiety Inventory
STAI-C: State-Trait Anxiety Inventory for Children
T: time point
VAS: Visual Analog Scale
VR: virtual reality

## Data Availability

Data sharing is not applicable to this article as no data sets were generated or analyzed during the current study.
