# Supplementary figures and images for: Comparing Smartphone Virtual Reality Exposure Preparation to Care as Usual in Children Aged 6 to 14 Years Undergoing Magnetic Resonance Imaging: Protocol for a Multicenter, Observer-Blinded, Randomized Controlled Trial
Source: JMIR Res Protoc. 2023 Jan 24;12:e41080. doi: 10.2196/41080 (PMC9906306; doi:10.2196/41080)

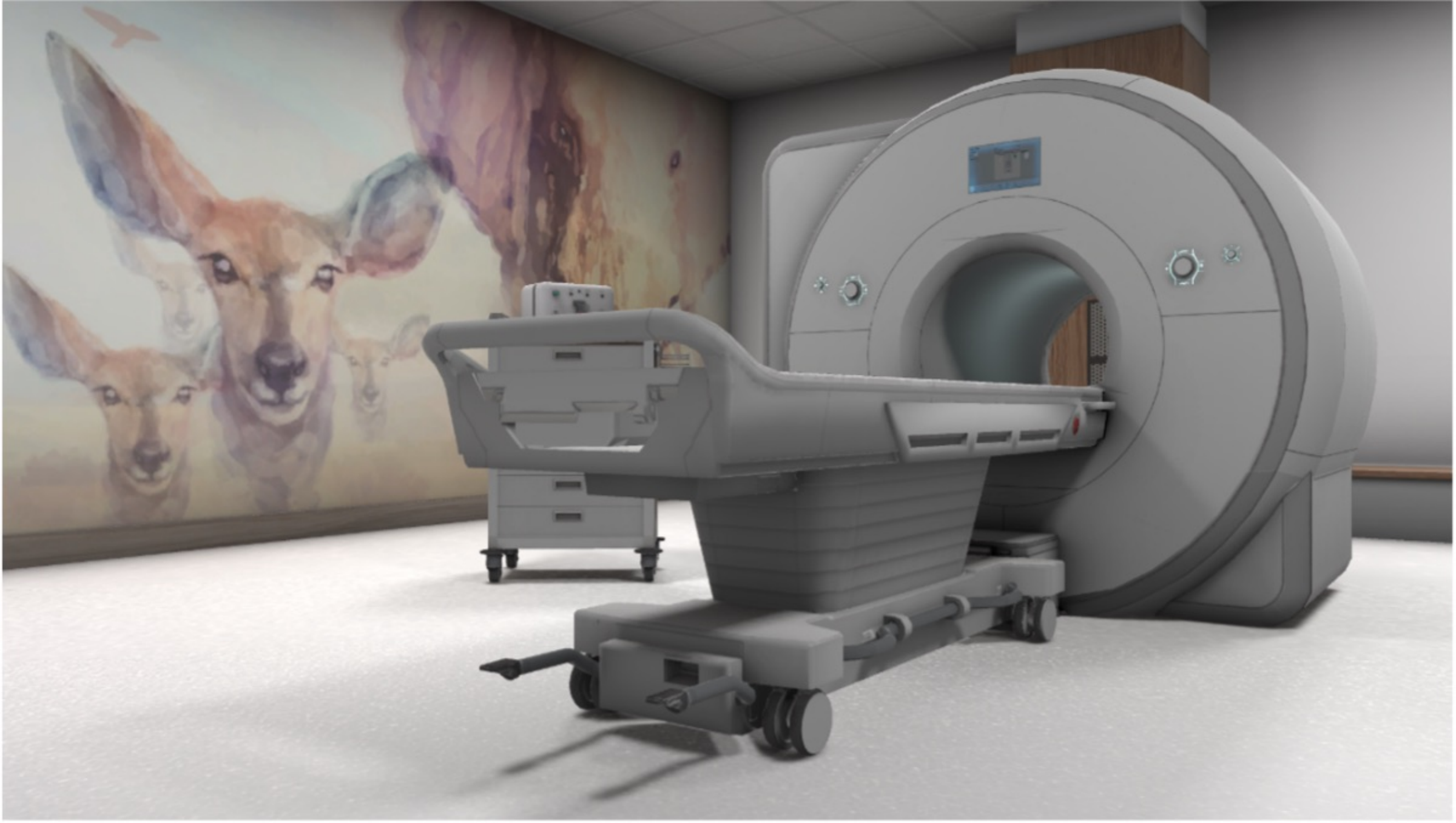

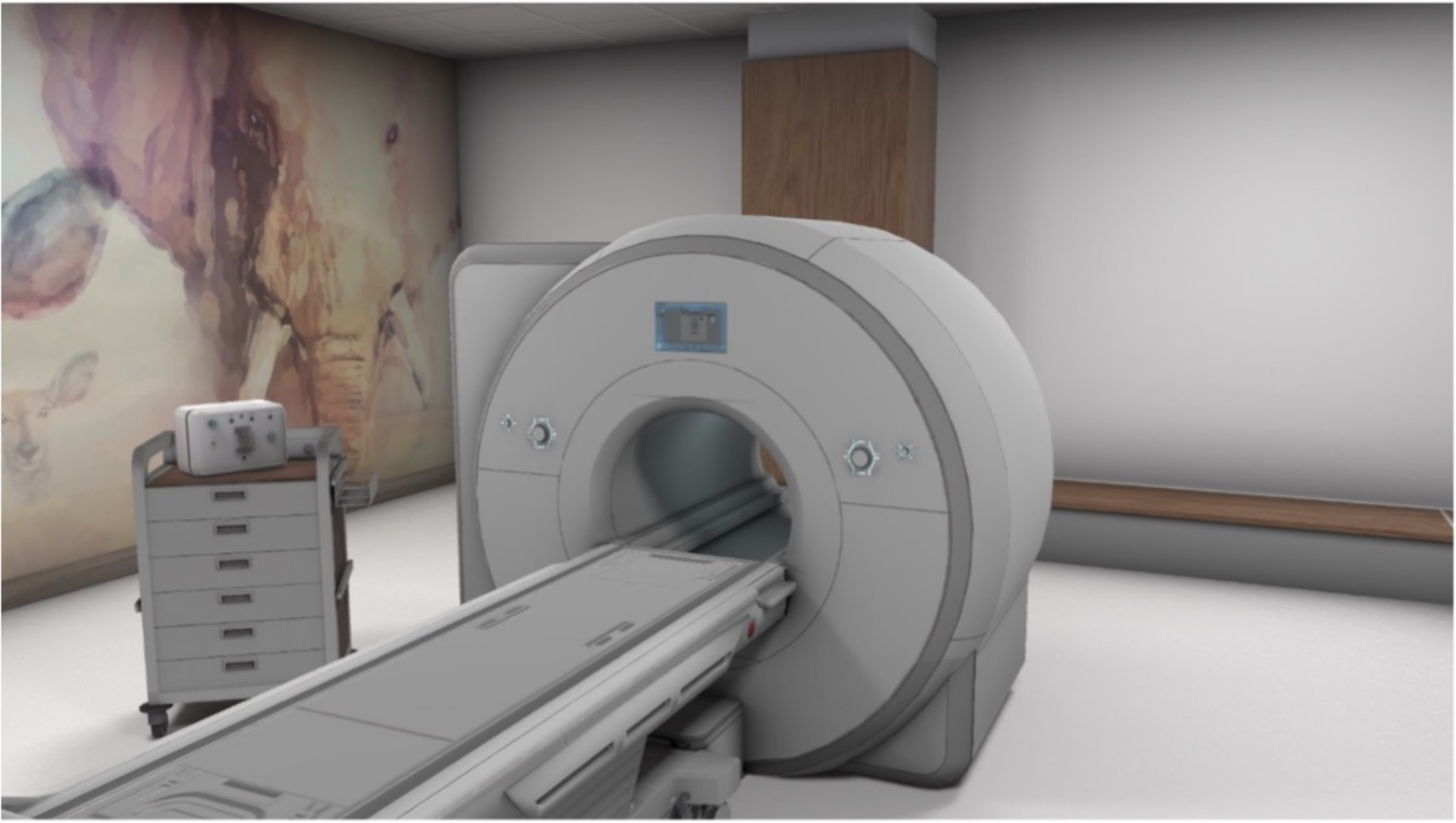

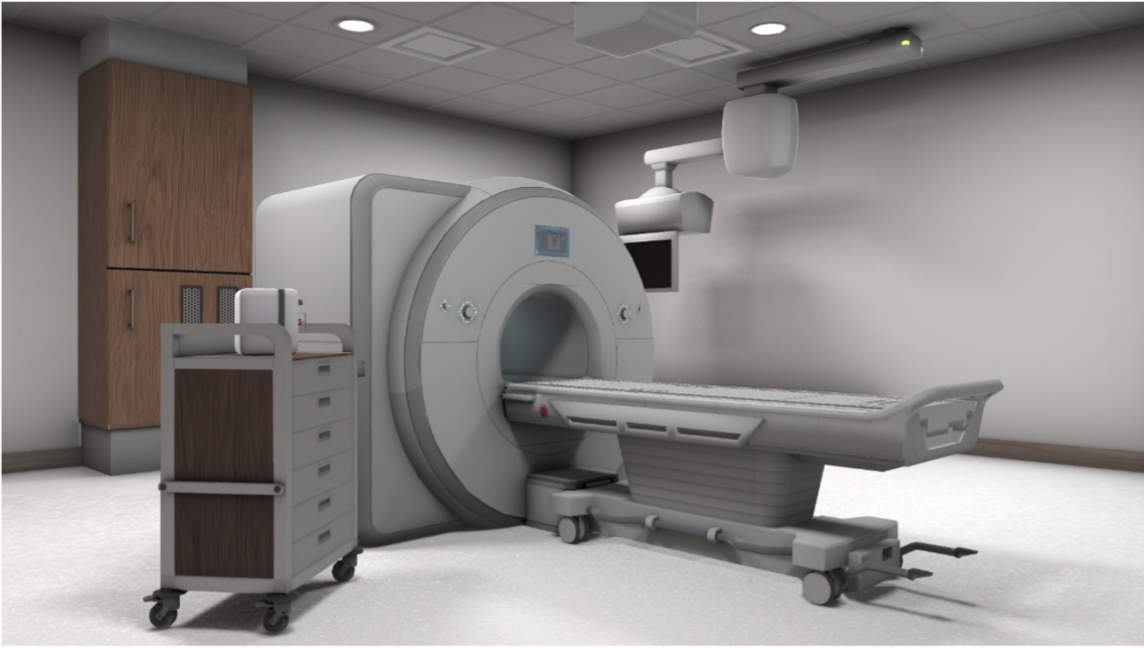

Supplement: Multimedia Appendix 1 [file resprot_v12i1e41080_app1.pdf]
